# Laparoscopic Management of Acute Small Bowel Obstruction: A Retrospective Study at a Tertiary Center in the United Kingdom

**DOI:** 10.7759/cureus.71089

**Published:** 2024-10-08

**Authors:** Atreya Subramanian, Christie Swaminathan, Jayas Siby, Anurag Singh, Adil Gulab

**Affiliations:** 1 Department of General Surgery, Aneurin Bevan University Health Board, Newport, GBR; 2 Department of Gastrointestinal Surgery, University Hospitals Sussex NHS Foundation Trust, Brighton, GBR; 3 Department of Digestive Disease and General Surgery, Royal Sussex County Hospital, Brighton, GBR; 4 Department of Gastrointestinal Surgery, Royal Sussex County Hospital, Brighton, GBR; 5 Department of General Surgery, Royal Sussex County Hospital, Brighton, GBR

**Keywords:** adhesions, emergency, laparoscopic, morbidity, mortality, small bowel obstruction

## Abstract

Objectives

To present a single-center experience of laparoscopic management of acute small bowel obstruction (ASBO) based on seven years of data and demonstrate its suitability for the United Kingdom (UK).

Methods

A retrospective review of case notes to evaluate postoperative outcomes was conducted. All emergency small bowel obstructions treated laparoscopically were included. The cases that were converted to a laparotomy were excluded. Demographics (age, sex), American Society of Anesthesiologists (ASA) grade, indication for surgery, duration of stay, complications, requirement of stoma, requirement of intensive treatment unit/high dependency unit (ITU/HDU), reoperation/readmissions, and 30-day mortality were noted. The results were tabulated and analyzed accordingly.

Results

There were 119 patients studied, with a median age of 66 (range: 17-97). The sex ratio was 62 females to 57 males. Primary etiologies of adhesion bands (49.5%, 59) and hernia (31.9%, 38) were the most common. Minor and major complications were 15 (12.6%) and 37 (31%), respectively. Three (2.5%) patients passed away within 30 days of surgery. The median length of stay (LOS) was eight days. The median LOS subgroup analysis showed nine days for adhesiolysis and six days for hernias.

Discussion

This study shows that there is significant heterogeneity in outcomes regarding small bowel obstruction around the world. We have demonstrated similar to better results in our center relative to other prominent centers in the UK. This can be attributed to the patient cohort, presentation, and physiological status on admission, delay to surgery, and associated co-morbidities to name a few.

Conclusion

This study indicates that laparoscopic surgery is a safe approach to treating ASBO, provided adequate expertise and infrastructure are available.

## Introduction

Small bowel obstruction (SBO) is one of the most common emergency scenarios surgeons face on a regular basis. According to a large data review by Szeliga and Jackowski, intraperitoneal adhesions (60-75%), cancers (20%), and hernias (10-20%) form the top three most common etiologies for SBO [1].

A meta-analysis by Krielen et al. in 2020 reported that laparoscopic surgery is not associated with better or worse postoperative outcomes compared with open adhesiolysis in the emergency setting. The study by Krielen et al. was specific only to adhesional obstruction, excluding all other etiologies [2].

The Bologna guidelines still recommend laparoscopic surgery wherever possible, but this is more with respect to preventing rather than treating adhesional small bowel obstruction. It also recommends non-operative management when possible when there are no clear signs for operative intervention (ischemia, peritonitis, etc.). Most importantly, there is an observation indicating that laparoscopic adhesiolysis may reduce postoperative morbidity, but there is no clear evidence as yet; results of randomized control trials (RCTs) are still awaited [3]. Hence, this study, even though retrospective (not an RCT), aims to add to the body of evidence in determining the efficacy and safety of adopting a laparoscopic first approach in the management of acute SBO.

A 2019 study comparing laparoscopic and open surgery in the management of acute small bowel obstruction (ASBO) demonstrated a lower complication rate and shorter stay among laparoscopically managed patients. However, the study did admit that the cohort managed laparoscopically was younger, had fewer previous surgeries, and was less complex in terms of operative difficulty and extent of adhesions [4].

There has been significant heterogeneity in the results comparing laparoscopic and open surgical management of ASBO. This is mostly attributed to the variations in patient population as well as expertise available. With evolving techniques and greater experience, we would expect a greater number of cases to be tackled laparoscopically. There is definitive proof that initial laparoscopic surgery reduces the incidence of adhesions in the future and subsequently lowers the incidence of bowel obstruction [5].

In the United Kingdom (UK), despite advances in laparoscopy in the emergency setting, there is still some reluctance to use these techniques as a universal standard starting point. A survey indicated that out of 384 respondents, only 200 (52.1%) consultants considered a laparoscopic approach for small bowel obstruction [6]. This is quite a surprising finding considering that laparoscopic surgery has become ubiquitous in the UK for most elective procedures.

In this study, we have examined the safety and feasibility of managing such cases of small bowel obstruction laparoscopically in a tertiary center in the UK. We have included a variety of etiologies, unlike most studies, which focus on adhesiolysis alone.

## Materials and methods

This was a single tertiary (and regional trauma) center retrospective study conducted using seven years of data ranging from February 25, 2016, to August 11, 2023.

Inclusion criteria

All emergency adult small bowel obstructions (categorized as code 1/2, i.e., immediate/urgent as per the National Confidential Enquiry into Patient Outcome and Death (NCEPOD) classification) who were operated on laparoscopically [7].

Exclusion criteria

All pediatric cases (aged <16 years) and procedures that were converted from laparoscopic to open operation were also excluded from the study. This was done as there would be an inherent bias in comparing this cohort’s (laparoscopic converted to open) mortality and morbidity statistics as they would naturally be either more challenging surgically or physiologically in a worse position necessitating open conversion.

Data source

This data was obtained by a combination of sources. The list of patients broadly fitting the necessary criteria was obtained from the trust’s central information unit. A review of case notes and discharge summaries of these patients was conducted from data available on the Trust IT system (Bamboo). Individual operative and case notes were studied to identify the exact procedure performed and to look for open conversion. Also, reoperations, if any, were also identified from the trusted software. Cases where ambiguity arose either in terms of diagnosis or future management were cross-verified with imaging from the trust picture archiving and communicating system (PACS) or histopathology/microbiology samples from the lab. Supplementary National Emergency Laparotomy Audit (NELA) data was also useful in cross-verifying details, including the timing of the surgery, as well as highlighting cases with higher predicted mortality and morbidity.

Data collection

Data was collected by three members of the team from the trust computers alone within the hospital premises. The data was also stored strictly only on trusted computers to maintain confidentiality. The parameters included in the study were basic demographics (age, sex), followed by the date of admission and date of surgery, as well as data on patient outcomes (ASA grade, 30-day mortality, morbidity, stoma formation, and ITU/HDU stay). Mutual agreement among members was reached regarding appropriate calculable data, namely duration of admission and complication rates.

Data analysis

A standard template was formed using a Microsoft Excel sheet, which included the previously mentioned parameters. Cases were grouped according to the type of procedure performed. All the results were further compiled in a master chart, segregating the data into etiologies (adhesions, hernias, and miscellaneous) to assess mortality, morbidity, etc. After due compilation, the results were presented at the monthly clinical governance meeting for approval.

Literature search

All standard electronic medical data on the incidence of specific etiologies, their mode of management, and their complications (mortality, morbidity) for the management of small bowel obstruction were obtained from PubMed-verified sources. Important definitions were obtained from other reputable sources, such as the web page for European emergency surgery and the official National Hospital Service (NHS) site for NCEPOD.

Data extraction 

All members involved in the study were in agreement regarding the manner and transparency in which the data was collected as well as the method in which the data was compiled and analyzed.

## Results

A total of 155 patients were retrieved over eight years (2016-2023) as per the records who had undergone an initial laparoscopic approach. About 36 patients (23.2%) were excluded from the study as there was open conversion, resulting in a final sample size of 119.

This comprised 58 patients undergoing adhesiolysis, 36 patients undergoing hernia repairs ± small bowel resection (all types of hernias were included, i.e., ventral, parastomal, incisional, etc.), and 25 patients classified as having miscellaneous etiologies. Nine were small bowel perforations related to Meckel's diverticulitis, followed by strictures. Five were due to Crohn's disease, and one was due to tuberculosis. Two were ischemic in origin, and one was due to intussusception.

The median age for the entire cohort was 66 years. The individual median ages for the groups involving adhesiolysis (group A), hernia repairs (group B), and miscellaneous etiologies (group C) were 69, 65, and 50.5 years, respectively. The overall gender ratio was 55 males to 64 females. The sex ratios for groups A, B, and C (male:female) were 22:36, 19:17, and 14:11, respectively (Table 1).

**Table 1:** Demographics

	Median age (years)	Sex ratio (male:female)	Total
Adhesions	69	22:36	58
Hernia	65	19:17	36
Miscellaneous	50.5	14:11	25

The ASA grading of groups A, B, and C was 2.49, 2.6, and 2.4, respectively. The complications were classified as minor (Clavien-Dindo score ≤ 2) or major (Clavien-Dindo score > 2). Group A had a total of 18 (31%) and nine (16%) minor and major complications, respectively. Group B had a total of 10 (27.7%) and three (8%) minor and major complications, respectively. Group C had a total of nine (36%) and three (12%) minor and major complications, respectively. The most common complication across segments was a surgical site infection followed by postoperative ileus. The most common major complication was pneumonia. Among groups A, B, and C, the number of patients needing ITU admission was nine (16%), three (8%), and one (4%), respectively. Only three deaths were noted, all of whom were in the adhesiolysis group (5%). Overall mortality was 2.5%. Two were due to sepsis (68 and 63 years of age at the time of death), and one was a postoperative myocardial infarction (85 years of age at the time of death) (Table 2).

**Table 2:** Mortality and morbidity findings ASA: American Society of Anesthesiologists; ITU: intensive treatment unit

	ASA	Minor complications	Major complications	ITU	30-day mortality
Adhesions	2.49	18 (31%)	9 (16%)	9 (16%)	3 (5%)
Hernia	2.6	10 (27.7%)	3 (8%)	3 (8%)	0
Miscellaneous	2.4	9 (36%)	3 (12%)	1 (4%)	0

The median duration of stay for groups A, B, and C were nine, six, and six days, respectively. The overall median duration of stay was eight days (Table 3).

**Table 3:** Duration of stay

Etiology	Median duration of hospital stay (days)
Adhesions	9
Hernia	6
Miscellaneous	6

Only two patients required a stoma to be formed; both were in group A.

## Discussion

A Chelsea-Westminister study conducted over 10 years regarding laparoscopic management of small bowel obstruction by Petrou et al. showed a mortality rate of 4.8% overall and 5.2% for patients managed exclusively by laparoscopy. Overall morbidity was 42.2% and 9.1% for minor and major complications. Our results were comparable, if not superior, considering a similar demographic of patients and available setup. The Chelsea study was intended to treat analysis and included the cases converted to an open procedure, which would intuitively have a higher complication rate, resulting in some level of discordance between the results [8]. Nonetheless, we have shown that laparoscopic management of these cases is safe and feasible with an acceptable conversion rate of 23.2%. Patients with an ASA of 3 and 4 showed a higher morbidity rate, including all three cases of mortality. Similar studies also show findings of a correlation between a higher ASA grade and complications (including conversion to a laparotomy).

The laparoscopic versus open adhesiolysis for adhesive small bowel obstruction (LASSO) trial conducted in Sweden in 2019, one of the few RCTs conducted on laparoscopic versus open adhesiolysis, demonstrated a longer postoperative stay as well as a higher complication rate (the complication rate was not statistically significant) in the open group relative to the laparoscopic group. This provides more empirical evidence favoring a laparoscopic first approach for small bowel obstruction [9].

A 2018 study conducted in South Korea involving 117 patients with small bowel obstruction treated laparoscopically demonstrated a shorter time to water/food intake as well as an overall shorter duration of hospital stay. A multivariate analysis was also performed and showed similar recurrence as well as complication rates with open surgery [10].

The National Acute Small Bowel Obstruction (NASBO) audit conducted in 2017 across the UK involved 2,434 patients presenting with ASBO of various etiologies. It demonstrated that among those patients undergoing operative intervention, a keyhole technique was used for only one in seven patients, of which half needed open conversion [11]. This is quite low compared to other studies in similar developed countries with comparable demographics. For example, a Danish study involving over 300 patients with ASBO demonstrated that one in two patients undergoing operative intervention was treated laparoscopically as the initial modality of treatment [12].

This study has shown a sufficient body of evidence in line with other prominent global studies indicating that laparoscopic surgery is appropriate for managing small bowel obstruction and has demonstrated both safety and feasibility. It has also shown that a laparoscopic approach for managing ASBO in a busy UK center is feasible and should be encouraged.

This was a retrospective observational study with a moderate sample size. The study focused on assessing the safety and feasibility of laparoscopic management of small bowel obstruction rather than comparison with open surgery. Confounding factors in this study could have been patient profile (number of previous surgeries, degree of peritoneal soiling, etc.), individual surgeon preference, and level of urgency. The ASA grade we noted did play a factor in determining the threshold for open conversion, but probably a more nuanced assessment would have been more appropriate, including the requirement of ionotropic/vasopressor support, a more detailed assessment of individual comorbidities, and scoring systems like NELA done preoperatively. All these factors, if considered, may provide a more robust study design. A more comprehensive study would involve an RCT similar to the LASSO trial but involving multiple etiologies rather than just adhesional obstruction.

The progression of any field must always be holistic and involve multiple dimensions. It is clear that laparoscopic surgery does have certain clear advantages when achievable, i.e., shorter hospital stay and similar to a lower complication profile. It also has been shown to reduce the chance of adhesions in the future. As experience and technology progress simultaneously, decision-making will lie in appropriate patient selection and having a reasonable threshold for open conversion guided by both surgeons and anesthetists. we should be able to reduce conversion rates. The bedrock of decision-making will lie in appropriate patient selection and having a reasonable threshold for open conversion guided by both surgeons and anesthetists.

## Conclusions

A laparoscopic first approach for most cases of ASBO is appropriate, provided patient stability and adequate case selection are ensured. Laparoscopy, whenever possible, does ensure superior outcomes and must be attempted when possible. Patient safety should always be kept paramount, and open conversion should not be delayed if the clinical situation demands. In the UK, there is probably a degree of apprehension in attempting many such cases laparoscopically. This could be attributed to training compulsions, busy emergency theaters, and adopting a faster, more familiar technique during unsocial hours, explaining the relatively lower rates of laparoscopically managed ASBO. We believe our study will encourage more surgeons in the UK to adopt a laparoscopic first approach to managing ASBO.
